# Comparative metabolomics of aging in a long-lived bat: Insights into the physiology of extreme longevity

**DOI:** 10.1371/journal.pone.0196154

**Published:** 2018-05-01

**Authors:** Hope C. Ball, Shiva levari-Shariati, Lisa Noelle Cooper, Michel Aliani

**Affiliations:** 1 Department of Anatomy and Neurobiology, Northeast Ohio Medical University, Rootstown, Ohio, The United States of America; 2 Musculoskeletal Biology Group, Northeast Ohio Medical University, Rootstown, Ohio, The United States of America; 3 Canadian Centre for Agri-Food Research in Health and Medicine, St. Boniface Hospital Albrechtsen Research Centre, Winnipeg, Canada; 4 Department of Foods and Human Nutritional Sciences, University of Manitoba, Duff Roblin Building, Winnipeg, Canada; Instituto Butantan, BRAZIL

## Abstract

Vespertilionid bats (Mammalia: Order Chiroptera) live 3–10 times longer than other mammals of an equivalent body size. At present, nothing is known of how bat fecal metabolic profiles shift with age in any taxa. This study established the feasibility of using a non-invasive, fecal metabolomics approach to examine age-related differences in the fecal metabolome of young and elderly adult big brown bats (*Eptesicus fuscus*) as an initial investigation into using metabolomics for age determination. Samples were collected from captive, known-aged big brown bats (*Eptesicus fuscus*) from 1 to over 14 years of age: these two ages represent age groups separated by approximately 75% of the known natural lifespan of this taxon. Results showed 41 metabolites differentiated young (n = 22) and elderly (n = 6) *Eptesicus*. Significant differences in metabolites between young and elderly bats were associated with tryptophan metabolism and incomplete protein digestion. Results support further exploration of the physiological mechanisms bats employ to achieve exceptional longevity.

## Introduction

**Fig 1:**
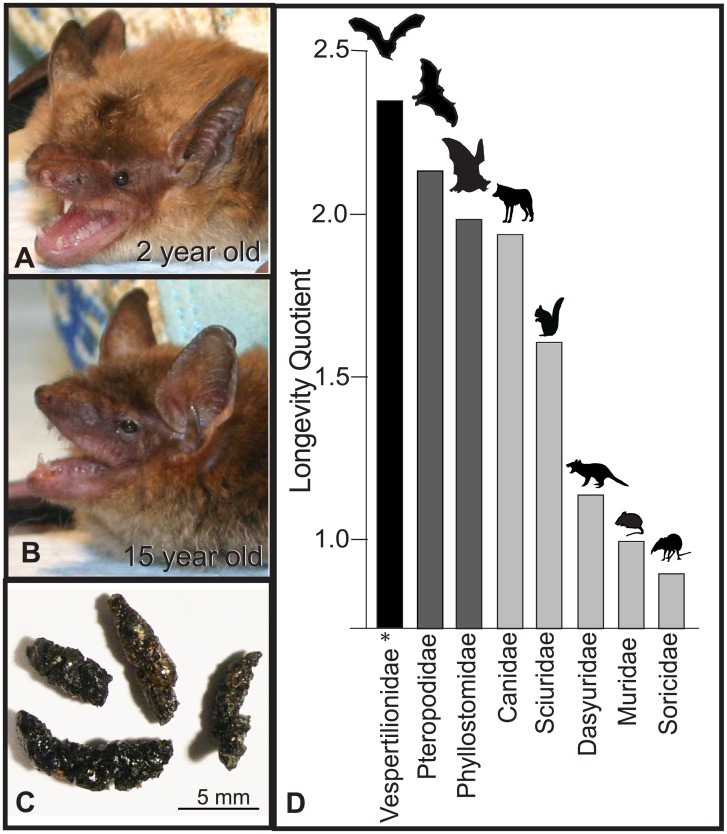
Vespertilionid bats, including big brown bats (*Eptesicus fuscus*), live 3-10x’s longer than equivalently-sized mammals but lack visible morphological characteristics indicative of age. *Eptesicus* may undergo a thinning of pelage hair throughout life as evidenced by a comparison of two-year old (A) and fifteen-year-old (B) adults. Longevity quotients across mammals (D, modified from [1,7]). Longevity quotient (LQ) = Observed/Expected longevity (from NFM regression) *Vespertilionid bar graph in D indicates longevity of *Eptesicus* (20-year lifespan) and excludes *Myotis* (over a 40-year lifespan) as it was not available for this study.

Studies of wild populations show that bats live longer than terrestrial mammals of comparable body size, and that the vespertilionid family of insectivorous bats displays extreme longevity [1,2] [Fig 1]. Notably, little- and big-brown bats (*Myotis* and *Eptesicus*) of the vespertilionid family, have a remarkable lifespan of 19–40+ years, a range that is 3–10 times longer than the expected longevity of size-comparable, non-volant mammals [3–6]. Most investigations into the mechanisms contributing to their extended lifespan are based on adult bats of unknown age as acquisition of known-aged animals requires long-duration banding-recapture studies or the maintenance of captive colonies over decades.

Physiological decline that occurs during the natural aging process ultimately leads to an increased prevalence of illness and exposes animals to heightened susceptibility to death. Age-accumulated cellular damage contributes to detrimental alterations that affect a plethora of processes [8] including intercellular communication [9,10], stem cell availability [11] and epigenetic regulation [12]. Cellular aging is partially the result of imbalances in concentrations of pro-oxidants and anti-oxidants [13] and the rate of aging is partially determined by the failure of protein homeostasis [14]. Protein turnover must accommodate challenges associated with oxidation, unfolding, and misfolding. Imbalances can result in the accumulation of insoluble proteins, as is the case in Alzheimer’s and Parkinson’s diseases [15]. Compared to other mammals, bats appear to have increased resistance to cellular damage and cellular aging. The mitochondria of *Myotis* are more efficient, per unit oxygen consumed, than those of similarly sized terrestrial mammals [16]. Bat liver proteins display increased resistance to oxidative stress and urea-induced unfolding associated with radiation exposure compared to mice [14]. Compared to carnivores and long-lived rodents, the fibroblasts of bats are more resistant to cellular stress when challenged with heavy metals, peroxides, and heat [17–19]. Bats also display increased expression of miRNA-155, a micro-RNA essential for defense against oxidative-induced cellular damage [20], and show positive selection of genes associated with reducing the negative effects of oxidation (e.g., *ATM*, *RAD50*, *KU80*, *MDM2* [21]). Furthermore, bats do not demonstrate loss of either protein homeostasis or muscle mass during hibernation [22]. Taken together, bats appear to lack novel modifications to protein repair, degradation and removal [14] and emphasize protein homeostasis.

Accumulation of genetic damage from exogenous and endogenous sources occurs during aging which negatively affects both nuclear and mitochondrial DNA [8,23]. The deleterious impact of this damage can include gene mutations [24], telomere shortening [25] and alteration in gene expression [26]. In bats, novelties in gene sequence and expression patterns probably promote genomic stability, prevent disease, and potentially contribute to their exceptional longevity. Evidence suggests bats promote genomic stability by preventing DNA damage to and pathologies associated with cancer. Specifically, bats display upregulation of genes associated with DNA repair (e.g., UVRAG), inhibition of cell proliferation and tumor formation (miRNA-16 and miRNA-143), and suppression of tumors (e.g., miRNA-101, *BRCA1*, *BRCA2*), as well as downregulation of a tumorigenesis promoter, miRNA-221 [20,21]. Furthermore, *Myotis*, like humans, do not express telomerase in either blood or fibroblasts and enhance DNA repair and prevent telomere shortening via expression of *ATM* and *SETX* genes [27]. Together, evidence suggests that bats uniquely promote genomic stability, DNA repair and cell proliferation with age to achieve exceptional longevity.

Research to explicate the mechanisms responsible for extending the health- and lifespan of bats expands our understanding of the natural aging process and the mechanisms used to delay senescence. Unfortunately, most research is comparative and biological innovations, such as unique miRNAs [20], identified in bats but not terrestrial mammals lack functional and phenotypic correlations. To fully understand the systematic and multi-scale approach bats use to combat aging and disease, scientists require access to known-aged animals. This study therefore addresses a critical gap in the field by utilizing a novel metabolomics approach to examine age-related differences in the fecal metabolomes of long-lived big brown bats (*Eptesicus fuscus*). By establishing the feasibility of utilizing a metabolomics approach to detect age-related differences in bat fecal samples, we lay the foundation for future studies to utilize this non-invasive approach to establish reference ranges in a larger cohort of animals and lay the groundwork for the utility of this method as an eventual means of age determination in bats.

Metabolomics measures low molecular weight compounds (metabolites) present in cells, tissues, or bio-fluids [28–30]. Examinations of excreted metabolites from known-aged bats may provide metabolic ‘snapshots’ of the changes that occur over the lifetime of a bat. When applied to fecal samples from wild-captured or captive-housed bats, metabolomics has the potential to provide insight into age-related differences in physiological processes. Previous metabolomics studies successfully identified age-related biomarkers for *C*. *elegans*, rodents, and humans [31,32] as well as biomarkers of human health [33,34]. Within bats, fecal metabolomics allowed for the study of the correlation between cortisol levels and environmental stress exposure in Isabelline Serotine bats (*Eptesicus isabellinus*; [35]), as well as track testosterone levels during the mating season, in a small, sac-winged bat (*Saccopteryx*; [36]). By utilizing fecal samples, these studies negated the need for collection of more invasive samples types, such as blood or teeth [36]. It could be that expansion of these methods to address age-related changes in excreted metabolites may eventually generate age-correlated candidate metabolites that offer insights into the mechanisms that uniquely extend the longevity of bats.

To provide insight into the age-related differences in the fecal metabolome of young and elderly adult big brown bats (*Eptesicus fuscus*) bats, this study utilized liquid chromatrography-quadrapole time-of-flight-mass spectrometry (LC-QTOF-MS) to compare the fecal metabolic profiles of long-lived *Eptesicus* (Fig 1). *Eptesicus* have a lifespan of approximately 20 years and our samples, taken from young (1–2 years) and elderly (14+ years) animals, analyzed variations in fecal metabolic profiles of samples separated by approximately 75% of this lifespan. Our work, although limited, significantly furthers the field by applying a metabolomics approach to show that analyses of fecal metabolic profiles can provide rapid, reliable, and non-invasive quantification of chemical biomarkers of aging. This ability to non-invasively compare differences in the fecal metabolome of young and elderly bats in a captive population lays the foundation for future experiments to apply metabolomics in a larger cohort of bats to identify age-specific biomarkers and elucidate specific signatures of senescence and aging mechanisms in bats. Moreover, fostering an understanding of physiological senescence in cardiac, skeletal, and sensory health in bats, may lead to a robust integration of aging and biomedical research.

## Material & methods

The Institutional Animal Care and Use Committee (IACUC) of Northeast Ohio Medical University approved the fecal sample collection under Protocol #17-06-142.

### Bats

The Cooper laboratory at Northeast Ohio Medical University (NEOMED; Rootstown, Ohio) maintains a known-aged colony of the big brown bat (*Eptesicus fuscus*; NEOMED IACUC #17-06-142) transferred from an original colony maintained by Dr. Ellen Covey at the University of Washington. While at the University of Washington, bats were banded according to year of capture and/or birth. These bats underwent natural hibernation and were exclusively fed an *ab libitum* diet of fresh water and meal worms (*Tenebrio molitor*) of known nutrient content [37]. Mealworm diet consists of non-organic bran supplemented with non-organic apple. These bats were transported to NEOMED and are housed indoors on a 12-hour light/dark artificial light cycle and fed the same *ab libitum* fresh water and meal worms diet [38].

### Longevity quotient estimation

The data utilized to determine LQ of selected mammal species (N = 139) were from a previously published dataset [7]. Predicted longevity was calculated for each species using a linear regression (slope = 0.172; y-intercept = 0.638) fitted to the logged values of body mass (g) and maximum longevity for all non-flying mammals (NFM) following published methods [1]. The longevity quotient was determined following Austad and Fisher [1] as: observed longevity/expected longevity.

### Fecal metabolomics

#### Fecal sample collection

Bats were isolated into individual cages overnight with *ab libitum* access to fresh water and meal worms. Feces (Fig 1C) were collected three times daily with each sample transferred to individual 1.5mL micro centrifuge tubes and stored immediately at -80°C. Fecal samples were acquired from a total of 28 individual bats ranging from 1 to greater than 15 years of age [1 year (n = 7), 2 years (n = 15), ≥ 14 years (n = 2), ≥ 15 years (n = 4)]. Although future work is aimed at establishing metabolic profiles for middle aged animals, at present, the sample size was too low to be included in this analysis. Samples were then shipped to the Nutritional Metabolomics Research lab at the Canadian Centre for Agri-food Research in Health and Medicine in Winnipeg, Manitoba at -80°C under the specifications of Canadian Food Inspection Agency import permit.

#### Extraction of metabolites

Frozen fecal samples were extracted, in duplicate, using a modified protocol adapted published protocols [39]. Bat fecal samples were thawed on ice. Aliquots (30mg) of each bat feces sample were mixed with 400 μl ultrapure water (Milli-Q H2O^®^, EMD Millipore, Billerica, Mass, USA) and homogenized for 1–2 min on ice using a Polytron PT 2600 ET Kinematica benchtop homogenizer (Fisher Scientific, Pittsburgh, Pa, USA). The probe was washed three times with distilled water between samples. After homogenization, samples were kept on ice while 3 μl of sodium azide (400μg/mL concentration; Sigma Aldrich, St. Louis, MO, USA) and 1 mL of ice cold methanol (Chromasolv, HPLC grade 99%; Sigma Aldrich, St. Louis, MO, USA) were added to each fecal sample. Samples were then vortexed for 30 seconds at 3000 rpm and then sonicated in a Branson^®^ 2800 ultrasonic bath (Branson Ultrasonics, Danbury, CT, USA). Samples were then centrifuged at room temperature in an Eppendorf 5415C centrifuge (Eppendorf, Hamburg, Germany) at 12621 rpm for 10 min. The resulting supernatant was removed and transferred to a clean 2 mL Eppendorf tube. One milliliter of ice-cold methanol was added to the remaining fecal sample and the mixture was vortexed for an additional 2 min at 3000 rpm. The sample was then centrifuged again as described above, supernatant removed and added to the first supernatant. The combined supernatants were then dried under a gentle stream of N_2_ and then stored at -80°C. Prior to LC-QTOF-MS injections, bat fecal sample extracts were reconstituted with 100 μl of acetonitrile: deionized water (4:1) and transferred into glass inserts in brown LC sample vials.

#### Metabolomics analysis

Analyses were conducted in duplicate using a Rapid Resolution HPLC system (1290 Infinity Agilent Ltd., Santa Clara, CA, USA) equipped with a binary pump, degasser, well-plate auto-sampler (maintained at 6°C throughout the runs) with a thermostat and thermostatic column. This system was coupled to a 6538 UHD Accurate LC-QTOF-MS (Agilent Technologies, Santa Clara, CA, USA) with dual electro-spray ionization (ESI) source. A 2.1 mm x 100 mm Agilent ZORBAX SB-Aq column (Agilent Technologies, Santa Clara, CA, USA) was maintained at 60°C for chromatographic separation of bat fecal samples. Two mobile phases were used, water (A) and acetonitrile (B), and both contained 0.1% formic acid. Run time for the reactions was set at 10 minutes with a gradient of 0–6 min 2% B; 6–8.50 min 60% B; 8.50–8.60 min 2% B and 8.60–10 min 2% B to re-equilibrate the column. A post-run time of 2 min was also instituted prior to the injection of the next sample. Sample carryover during successive injections was minimized by washing the needle in two separate vials of mobile phase (5 washings per sample vial) before each new sample injection. For each individual sample analysis, 3μl of fecal extracts was injected with a flow rate maintained at 0.7 mL/min.

MS data acquisitions were completed in both positive (+) and negative (-) electrospray ionization (ESI) modes. MS parameters included capillary voltage (4000 V), the fragmentor (175V), the skimmer (50V) and the OCT 1 RFVpp (750 V). For drying, nitrogen gas (N_2_) was utilized at 11 L/min at 300°C with nebulizer settings at 50 psig. MS spectra were collected within the range of 50–1700 m/z and known references masses of 121.0508 and 922.0097 (ESI+) and 112.9860 and 1033.9880 (ESI-) utilized during all runs.

#### Data processing

The LC-QTOF-MS metabolomics data workflow followed standard protocols (Table 1) and utilized multiple algorithms incorporated in Agilent MassHunter Qualitative (MHQ, 7.01 and Mass Profiler Professional (MPP, version 12.6.1) software programs. Raw chromatographic files, total ion chromatograms (TICs), generated during LC-QTOF-MS runs were acquired and stored as “*.d” files for MHQ data processing. Raw chromatographic files were first processed using both Molecular Feature Extraction (MFE) to detect features with abundances ˃ 4000, and find by formula (fbf) algorithms in MassHunter Qualitative (MHQ) software (Agilent Technologies, Santa Clara, CA, USA) to generate chemical formulae, based on exact masses and annotate detected metabolic entities. The method also provided information regarding [M + H] +, isotope distribution of each candidate molecule and any corresponding sodium adducts. Extracted ions were now considered single features and MHQ algorithms were used to generate potential identifications via Metlin [40] and MHQ/MPP-associated software libraries utilizing chemical formulae. These features, with their potential chemical formulae, associated retention times (RT), exact masses and ion abundances were then converted into compound exchange format (“*.cef”) files. These “.cef” files were exported to MPP once more as part of recursive analyses. Individual “*.cef” files were now binned into the two-designated age groups (1-2-year-old “young” and 14-15-year-old “elderly” individuals). These were then combined, aligned and normalized before application of the ‘Find by ion’ algorithm and subsequent generation of new “*.cef” files. These newly generated “*.cef” files were exported to MHQ for additional data mining. Targeted feature algorithms were also applied to minimize the risk of false positive and false negative features. Another set of individual “*.cef” files were then generated from the original “*.d” files and exported into MPP for statistical analyses. To ensure that potential feature extraction artefacts were not eliminated, features which were detected in a minimum of one condition were excepted using frequency filtration and other MPP filtration methods such as: included charge states set to “all charge states permitted” and number of detected ions was set at “2”. Retention time (RT) compound alignment parameters were set to 0.15 min and the mass tolerance was set at 2.0 mDA. Data were normalized using a percentile shift algorithm (set at 70) and baselined to the median of all samples.

**Table 1:** Summary of the workflow utilized to investigate the metabolic profile of feces in young and elderly bats.

**Step 1: Metabolomics of Bat Fecal Matters (LC-QTOF-MS and MHQ 7.01)**	
a)	Non-targeted analysis of bat fecal samples by LC-QTOF-MS in positive and negative ESI modes.
b)	Molecular Feature Extraction (MFE) algorithm to extract all detectable compounds.
c)	Find-by-Ion algorithm to remove false positive and negative compounds.
**Step 2: Statistical Analysis (MPP 12.6.1) to Compare Young and Elderly Metabolomes**	
a)	Mann-Whitney U-test (P < 0.05) followed by robust multiple testing correction of p-values (Benjamini Hochberg FDR multiple testing).
**Step 3: Biochemical Pathway Analysis—Assignment of Metabolite Function**	
a)	Identification of biochemical pathways (KEGG, Human Metabolome Database, Metlin) for the metabolites present in fecal matters of young and old bats and their potential correlation with age.

Metabolic entities were identified using the integrated Metlin database, which contains greater than 200,000 known compounds, within the MHQ and MPP software. Compounds that were not matched with those available in the Metlin database were listed as “endogenous metabolites” and will be the subject of future studies using liquid chromatography-nuclear magnetic resonance (LC-NMR). Because a goal of the study was identification of potential candidates characteristic of an elderly metabolome, candidates were selected that demonstrated significant concentration increases in elderly fecal samples. Common databases such as KEGG [(41)], Human Metabolome Database [41] and published literature were mined to assign potential physiological functions.

#### Statistical analyses

Statistical analyses (Table 1) included Mann-Whitney U-test (p <, 0.05) with Benjamini Hochberg FDR multiple testing correction. Principle component clustering models (PCA) were constructed in MPP software (version 12.6.1). These statistical analyses of the original identified candidates identified 42 that demonstrated significant (p < 0.05) age-related increases or decreases in concentration between the young and elderly bats (Table 2).

**Table 2:** Fecal analyses detected 41 metabolites with known biological effects that significantly differentiated metabolome of elderly bats. Metabolites demonstrated ≥2-fold changes and p < 0.05. Log_2_ abundance ratios for all metabolites were acquired in ESI+ mode.

Tryptophan Metabolism and Oxidation							
	*Metabolite*	*Formula*	*Non-normalized abundance [young]*	*Non-normalized abundance [elderly]*	*Log2 abundance ration ([elderly]/[young])*	*p (Corr)*	*Reference*
1	3-Amino-2-naphthoic acid	C_11_ H_9_ N O_2_	2.3	229712.5	16.6	0.01010	[42]
2	Indole	C_8_ H_7_ N	1.3	4025.5	11.6	0.01010	[43–46]
3	3-Methylindole	C_9_ H_9_ N	0.9	704.8	9.6	0.01010	[47,48]
4	2-aminomuconic acid semialdehyde	C_6_ H_7_ N O_3_	0.9	457	9.0	0.01147	[49]
5	N, N-Dihydroxy-L-tryptophan	C_11_ H_12_ N_2_ O_4_	2.2	477.8	7.8	0.01010	[49]
**Incomplete Protein Digestion/Catabolism**							
6	Threoninyl-Phenylalanine	C_13_ H_18_ N_2_ O_4_	1.9	19997.9	13.3	0.01010	[50]
7	Valyl-Isoleucine	C_11_ H_22_ N_2_ O_3_	4.2	9355.4	11.1	0.01010	[50]
8	Leu Asp Lys	C_16_ H_30_ N_4_ O_6_	3.1	4252.3	10.4	0.01108	[50,51]
9	Prolyl-Lysine	C_11_ H_21_ N_3_ O_3_	1.7	1426.9	9.7	0.01010	[50]
10	Ile Gly Arg	C_15_ H_24_ N_10_	0.9	396.2	8.8	0.01010	[50]
11	Pyrrolidine	C_4_ H_9_ N	4.1	1580.7	8.6	0.01010	[50]
12	Pro Val Pro	C_15_ H_28_ N_4_ O_4_	1.3	320.2	8.0	0.01010	[50]
13	Leu Lys Ala	C_21_ H_32_ N S	1.3	263.7	7.6	0.01010	[50]
14	Leu Pro Lys	C_18_ H_28_ N_8_	1.3	233.3	7.5	0.01384	[50]
15	Histidinylglycine	C_8_ H_12_ N_4_ O_3_	1.9	239.1	6.9	0.01010	[50]
**Protein and Amino Acid Synthesis**							
16	L-Homocitrulline	C_7_ H_15_ N_3_ O_3_	26.2	17837.7	9.4	0.01147	[52]
17	L-Lysine 1,6-lactam	C_6_ H_12_ N_2_ O	2.1	316	7.3	0.01157	[49]
18	Ganglioside GD3 (d18:0/12:0)	C_64_H_113_N_30_O_29_	1.4	186.8	7.1	0.01751	[41]
**DNA degradation**							
19	N2,N2-Dimethylguanosine	C_12_H_17_N_5_O_5_	0.9	243.9	8.0	0.01157	[53]
**Glycation Indicator**							
20	N2-Fructopyranosylarginine	C_12_ H_24_ N_4_ O_7_	2.2	700.1	8.3	0.03993	[54]
**Mitochondrial Respiratory chain failure**							
21	Malonylcarnitine	C_10_ H_17_ N O_6_	5.3	3095.4	9.2	0.01572	[55]
**Fatty Alcohol**							
22	2,4,6-Octatriyn-1-ol	C_8_ H_6_ O	23.2	79422.5	11.7	0.01157	[41]
**Biotin Metabolism**							
23	apo- [3-methylcrotonoyl-CoA: carbon-dioxide ligase (ADP-forming)]	C_7_ H_15_ N_3_ O_2_	5.7	5340.6	9.9	0.02793	[41]
**Methane Production**							
24	N-Furfurylformamide	C_6_ H_7_ N O_2_	21.5	22187.8	10.0	0.01010	[56]
**Cytoketogenesis**							
25	Isopentenyladenine	C_10_ H_13_ N_5_	2.8	1624.2	9.2	0.04454	[57,58]
**Isothiocyanate**							
26	6-Isothiocyanato-1-hexene	C_7_ H_11_ NS	1.4	990.5	9.4	0.01108	[41]
**Organofloride**							
27	2-Chloro-1,1,1-trifluoroethane	C_2_ H_2_ Cl F_3_	1.3	206.1	7.3	0.01010	[41]
**Bacteria Quorum Sensing/ Biofilm inhibition**							
28	N-3-oxo-tetradec-7(Z)-enoyl-L-Homoserine lactone	C_18_ H_29_ N O_4_	1.8	137.7	6.2	0.01157	[59]
**Fructosylation Product**							
29	N-(1-Deoxy-1 fructosyl) threonine	C_10_ H_19_ NO_8_	1.9	209	6.7	0.025300	[41]
**Antibiotics/Pharmaceuticals**							
30	Lomustine	C_9_ H_16_CI N_3_ O_2_	2	482.3	7.9	0.01157	[60]
31	Methylmercury chloride	C H_3_ Cl Hg	2.2	465.6	7.8	0.01010	[61]
32	Caldine	C_26_ H_33_ NO_6_	1.4	286.5	7.7	0.01010	[62]
33	Gaboxadol	C_6_ H_8_ N_2_ O_2_	2.1	366.5	7.4	0.02525	[63]
34	2-Propylglutaric acid	C_8_ H_14_ O_4_	0	5.7	9.6	0.01239	[64]
35	Istamycin X0	C_14_ H_30_ N_4_ O_4_	1.4	199.6	7.2	0.02793	[49]
**Pesticides/Fungicides**							
36	Bithionol	C_12_ H_6_ Cl_4_ O_2_ S	1.7	118.4	6.1	0.01157	[65]
37	Zinnimidine	C_15_ H_19_ NO_3_	1.9	277	7.2	0.01239	[66]
38	Dimethyl phosphate	C_2_ H_7_ O_4_ P	1.1	152.9	7.1	0.01010	[67]
39	Procymidone	C_13_H_11_Cl_2_NO_2_	1.5	1499.3	9.9	0.01010	[68,69]
**Endogenous Peptides**							
40	1-amino-3,3-diethoxypropane	C_7_ H_17_ N O_2_	4.1	1812.9	8.8	0.01157	
41	α-Thiophenecarboxylic acid	C_5_ H_4_ O_2_ S	2.8	1018.4	8.5	0.01010	

## Results

### Metabolites

Our metabolomics analysis included 28 *Eptesicus*, ranging from one to over fourteen years of age. To identify age-associated differences in metabolic profiles, the animals were divided into two different age groups: the “young” group included 22 animals aged 1–2 years, while the “elderly” group contained 6 individuals all 14 years of age and older. The final metabolite dataset contained 2003 entities (unidentified atoms, molecules, compounds and/or compound fragments present in both age groups. Resultant identified metabolites therefore generated a pilot list of candidate metabolites that set the stage for larger age-comparative and physiological studies.

**Fig 2:**
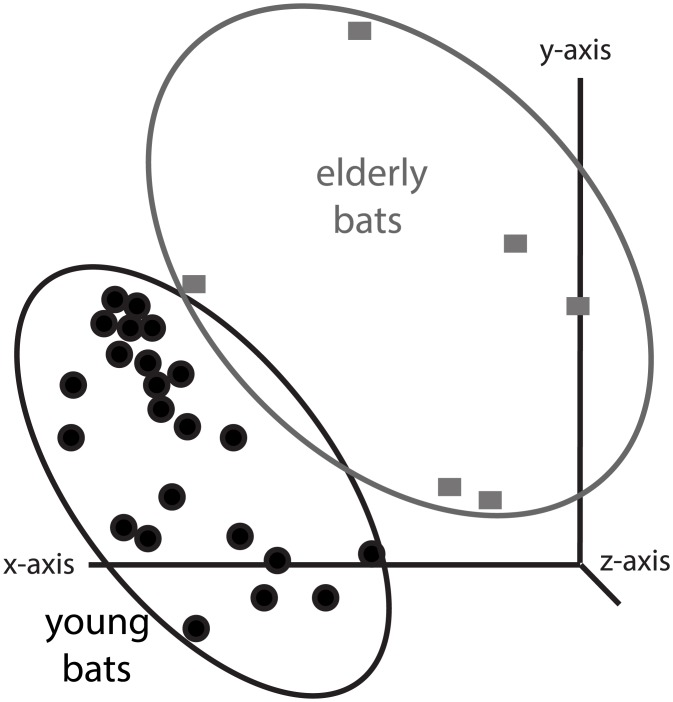
Principle component analysis (PCA) of metabolic profile of fecal samples show separate groupings for age groups. The young age group is demonstrated by open squares (one-year-old animals) and, filled, black squares (two-year-old animals). The elderly age group is demonstrated by open circles (fourteen-year old animals) and filled, black circles (fifteen-year-old animals). Variation was explained on three axes: x-axis (18.82%), y-axis (7.98%), and z-axis (5.69%).

Forty-one compounds within the feces displayed significantly higher concentrations (p < 0.05) in the elderly metabolome (Fig 2, Table 2). Roughly 20% of these compounds are associated with protein and amino acid metabolism (Table 2), where significant increases with age suggest decreases in incomplete protein catabolism and/or absorption. Of these compounds found in the elderly metabolome, the most abundant are dipeptides (i.e., threoninyl-phenylalanine (p = 0.01010), valyl-isoleucine (p = 0.01010), prolyl-lysine (p = 0.01010), and histidinyl-glycine (p = 0.01010)) and tripeptides (leucyl-aspartyl-lysine (Leu Asp Lys; p = 0.01108), isoleucyl-glycyl-arginine (Ile Gly Arg; p = 0.01010), prolyl-valyl-proline (Pro Val Pro; p = 0.01010), leucyl-lysyl-alanine (Leu Lys Ala; p = 0.01010), leucyl-prolyl-lysine (Leu Pro Lys; p = 0.01384)). During normal metabolism in other mammals, these peptides can be either cleaved by proteases, or absorbed by the enterocytes of the intestinal walls via transcytosis or co-transport with ions (e.g., H^+^, Na^+^; [47]). Comparisons of digestive efficiency in bats and non-flying mammals showed a similar digestive efficiency for proteins when consuming meal worms [50], which is the diet of our study taxon. Therefore, an increased abundance of di- and tripeptides within the feces of elderly bats may indicate that bats undergo a decline in peptide degradation capacity that may be associated with digestive senescence [70].

**Fig 3:**
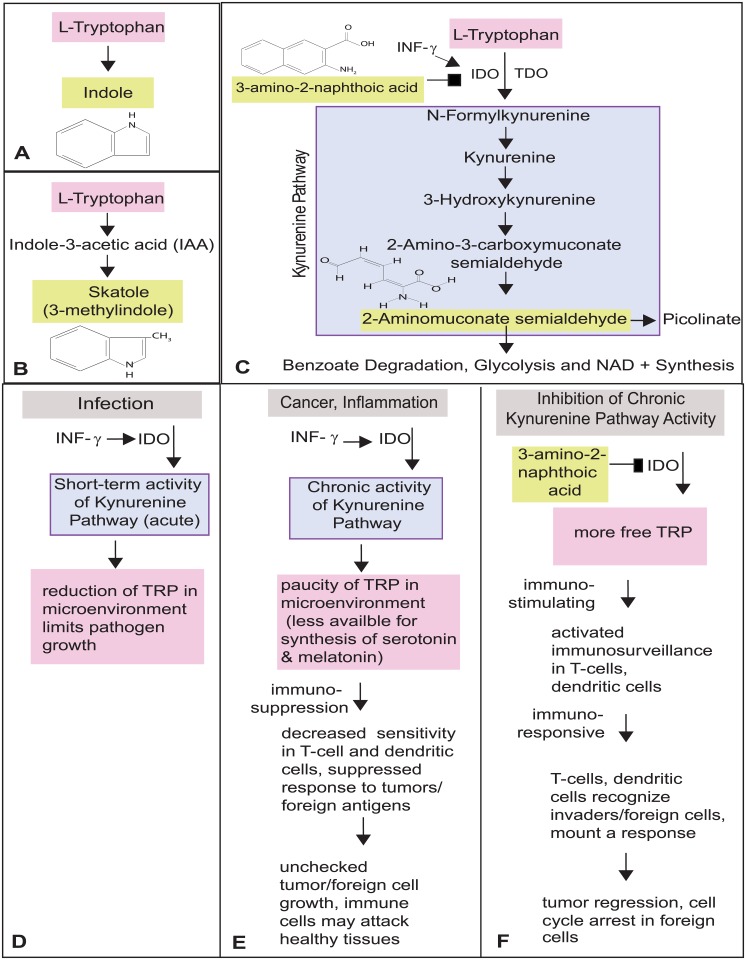
Metabolism of L-Tryptophan can occur as a direct conversion to indole (A), modification to skatole (B), and degraded by the kynurenine pathway (C). IDO is the most critical enzyme regulating local activity of the Kynurenine Pathway. In response to an infection (via IFN-γ), activation in IDO results a local degradation of tryptophan that limits growth of a pathogen (D). Activation of IDO in cases of chronic inflammation/cancer suppresses the effectiveness of immune cells which creates a sanctuary for pathogen survival and proliferation (E). In contrast, inhibition of the IDO response results in the stimulation of immune cells and catalyzes an immune response that may arrest growth and hinder proliferation of pathogens (F). Metabolites with high concentrations in the feces of elderly *Eptesicus* are indicated by yellow boxes.

Of the candidate metabolites, four were functionally associated with metabolism of the amino acid tryptophan (Fig 3; indole (p = 0.01010), 3-Methylindole (skatole; p = 0.01010), 3-amino-2-naphthoic acid (p = 0.01010), and 2-aminomuconate semialdehyde (p = 0.01147)). These compounds displayed some of the highest concentrations of recovered metabolites from the elderly samples, suggesting their concentrations increase with age in *Eptesicus*. This finding was striking as some of these compounds are known to contribute to longevity and play key roles in immune function including cancer and inflammation [46,71,72].

Several synthetic compounds of non-biological origin were also upregulated in elderly bats, but at lower concentrations compared to those functionally associated with tryptophan and protein metabolism. These compounds included an anti-convulsant (2-Propylglutaric acid (p = 0.01239); [64]), an anti-cancer compound (Lomustine (p = 0.01157); [60]), an anti-hypertensive (Caldine, also known as Lacidipine (p = 0.01010); [62]), and an anti-tapeworm and anti-algal metabolite (Bithionol (p = 0.01157); [65]). One potential source of many of these synthetic compounds may be the meal worm food source. The meal worms are raised on a non-organic diet, potentially exposing them to a wide range of chemical contaminants. Future work examining the metabolic profile of the meal worms themselves is planned to test this hypothesis. Evidence of these compounds recovered from feces may indicate impaired detoxication in bats resulting in retention of these compounds.

Beyond skatole and peptides, still other compounds (Table 2) potentially shed light on the physiology of age-related declines in bats. It could be that these compounds offer insight into the long-standing questions of what causes long-lived bats to eventually die. Samples recovered from elderly bats showed increased concentrations of N2-Fructopyranosylarginine (p = 0.03993), a precursor to advanced glycation end products (AGE), which are detrimental accumulations of glycated and oxidized proteins affecting diverse systems like connective tissue integrity, renal function, and aging [54]. The presence and increased concentrations of these compounds in samples from elderly *Eptesicus* potentially indicate the onset of glycation. Although multiple studies showed modifications associated with mitochondrial function [16,20], results of this study showed increased concentrations of a potential indicator of mitochondrial deficiency in elderly bat samples (Malonylcarnitine; p = 0.01572; [55]). Presence of the nucleoside N2, N2-Dimethylguanosine (p = 0.01157) potentially indicates upregulation of transfer RNA (tRNA) degradation [53]. Presence of these metabolites are suggestive of age-related declines in bat health although further study is required to acquire definitive evidence.

## Discussion

The exceptional longevity of many bat species has piqued the interest of researchers aiming to elucidate the physiological adaptations contributing to these extended lifespans [14,19,21]. This study tested the feasibility of a comparative fecal metabolomics approach in young and elderly big brown bats (*Eptesicus fuscus*). Analyses detected 41 metabolites that demonstrated significant increases with age in *Eptesicus*. Currently, our analyses lack a larger cohort of elderly animals as well as middle-aged samples which, unfortunately, impedes understanding of how these metabolites do or do not fluctuate across the lifespan in *Eptesicus*. Future studies will target larger sample sizes of young, middle, and elderly age groups to better assess how fecal metabolites vary with age and better understand the range of individual variation within these animals. Beyond offering the first age-comparative study of fecal metabolomics in bats, our results also provide new insights into how bats maintain physiological health into old age.

Although other tissues and fluids are available for metabolomics analysis (e.g., hair, blood, urine), feces were the focus of this pilot analysis as they offer a reliable and non-invasive means for sample collection that may ultimately be useful for data collection in wild populations. We utilized databases containing identities for over 200,000 metabolites to analyze our fecal samples and 41 metabolites that demonstrated age-associated differences between young and elderly bats. While urine is commonly utilized in conjunction with fecal analyses, the authors found collection of uncontaminated urine to be a major obstacle. Future assays will also address how circulating levels of identified metabolites in blood correlate to levels found in bat fecal samples.

### Protein homeostasis and aging

Progressive deterioration of protein homeostasis contributes to age-related functional decline and adversely affects longevity. The cellular mechanisms of translation, folding and clearance cooperatively regulate protein production and stability as well as the refolding or removal of damaged products [73]. With age, incomplete breakdown of proteins becomes more common in mammals [74,75], and it appears to occur in elderly bats as well with increased levels of di- and tripeptides detected in elderly fecal samples. Reactive oxygen species (ROS) are a leading contributor to oxidative protein damage [75], resulting in accumulation of abnormal proteins associated with numerous pathological conditions [76]. Recent evidence suggests that bats possess increased resistance to oxidative stress [14,20], which assists in maintaining protein homeostasis. Heat shock proteins (HSPs) are molecular chaperone proteins induced in response to stress and other stimuli that are critical to the maintaining protein quality [77]. Synthesis of HSPs is impaired in aging in many mammals, including humans [78]. Vespertilionid evening bats (*Nycticeius humeralis)* and little brown bats (M*yotis lucifugus*)) have increased HSP levels compared to shorter-lived rodents and marsupials [19]. These elevated chaperone levels may support a healthy stress response [79] and increase longevity in some bat species. Additionally, bats possess lower levels of both protein breakdown and removal and demonstrate an increased resistance to urea-induced protein unfolding compared to mice [14]. Our results suggest elderly bats experience age-related declines in peptide degradation capacity and peptide absorption similar to that seen in other mammals.

### Tryptophan metabolism and its influence on longevity

The fecal microbiome is an ecosystem of over 1000 species of bacteria which undergo complex interactions that ultimately influence the physiology and health of their host. Metabolites formed during the bacterial metabolism of the essential amino acid tryptophan (TRP) play key roles in the molecular regulation of host behavior, neuronal activity, immune activity, longevity and extend the healthspan [46,80]. Within our sample of metabolites that demonstrated age-related differences between the young and elderly microbiome, four metabolites play a role in the metabolism of tryptophan (i.e., indole, skatole, 3-amino-2-naphthoic acid, 2-aminomuconate semialdehyde, N, N-dihydroxy-L-tryptophan; Fig 3). Of these, the tryptophan metabolism product indole is the most well-understood. Indole is used as a biomarker indicative of 85 species of gram-negative and gram-positive bacteria [44] and functions to enhance the intestinal barrier [43], support dynamic signaling within microbial communities [81], as well as act as an anti-inflammatory molecule in cell cultures [45] as well as increased healthspan [46]. Because indole is the most prevalent tryptophan degradation product, our results suggest the increased abundance of indole within intestines of elderly bats may function to offset age-related stressor and declines in intestinal health and microbiome-related diseases [46,82] and potentially increase the healthspan of elderly *Eptesicus*.

The most common naturally occurring analog of indole is skatole (3-methylindole; [48]), also known as the “essence of poop” based on its smell in high concentrations (Fig 3). Although typically found in much lower concentrations than indole, skatole serves as a biomarker for colon cancer [83] and is known to induce kidney damage, pulmonary edema, emphysema, and death in livestock [84]. Skatole also adversely effects biological membranes and promotes the destruction of red blood cells (hemolysis; [47]. Within our sample, elderly bats displayed a marked increase in skatole concentrations. Because skatole is a biomarker for intestinal health [83,85], age-related increases in its concentration in elderly bats suggest some form of physiological decline within the intestines. However, this is not straightforward because elderly bat feces also contain high concentrations of indole, a metabolite that promotes intestinal health.

Tryptophan is most commonly metabolized via the kynurenine pathway (Fig 3), which generates metabolites affecting energy metabolism, neurodegeneration, immune regulation [80], inflammation [86] and bone diseases [87]. Tryptophan degradation along the kynurenine pathway results in increased concentrations of kynurenine and its derivatives (collectively referred to as kynurenines) intracellularly or within the microenvironment [88]. With age, humans increase the rate of tryptophan degradation resulting in higher concentrations of kynurenines relative to tryptophan, a process thought to contribute to aging [89]. Within elderly bats, fecal metabolites showed evidence of the kynurenine pathway of tryptophan degradation. A metabolite nested within the pathway (2-aminomuconate semialdehyde) was increased in elderly bats as were concentrations of 3-amino-2-naphthoic-acid, a molecule that modifies the rate of tryptophan degradation within the kynurenine pathway [42]. Interestingly, the metabolomic profiles of bat feces suggest they may lack this progression toward increased concentrations of kynurenines with age.

The first step of the kynurenine pathway, the breaking of the indole ring of L-tryptophan, is regulated by the enzymatic activities of indoleamine 2, 3-dioxygenase (IDO) and tryptophan 2,3-dioxygenase (TDO; [80]). Normally TDO and IDO act in tandem, but their actions become unsynchronized under pathological conditions [42]. Whereas TPO produces most kynurenine within the blood circulation, IDO is expressed in many cells throughout the body (e.g., mesenchymal stem cells, macrophages) to regulate local tryptophan concentrations. We focus our discussion on IDO because it bears the most relevance to the fecal metabolites of bats. Depending on conditions within the microenvironment, activation of IDO can have beneficial and pathological consequences (Fig 3). During an acute infection, short-term activation of IDO induces a local depression in the concentration of tryptophan, which starves pathogens of this amino acid, thereby reducing the capacity for pathogenic growth and making them more susceptible to cell-cycle arrest and apoptosis [90]. This acute interaction is largely beneficial to the host. However, in response to continuous cytokine production as in chronic inflammatory disease and cancer, the pathogen is not removed from the microenvironment. In this case, long-term or chronic exposure of IDO results in a build-up of kynurenines that modify amino acid and metabolite sensors on antigen-presenting cells (e.g., dendritic cells, T-cells). In response to a lack of sensitivity, these cells fail to recognize tumor cells and therefore tolerate their presence. Tumor/pathogen growth proceeds unchecked by the immune system [80]. In addition, some immune cells instead attack healthy cells of the host and ultimately contribute to age-related and inflammation-related diseases (e.g., sarcopenia, dementia, diabetes; [91]). As such, the kynurenine pathway is a major focus of immunological research.

In contrast, inhibition of IDO promotes immune cell health and activity (Fig 3). Inhibition of IDO results in a greater number of tryptophan molecules within the local microenvironment that are free to be metabolized in other essential pathways influencing behavior and reproduction (serotonin; [92]) and circadian rhythms (melatonin; [93]). Relieved of IDO-dependent suppression, immune system function is improved as T-cells and dendritic cells are sensitized and able to detect the presence of tumor cells/pathogens [94,95]. Activity of these cells ultimately allows for an effective immune response that causes tumor regression via cell-cycle arrest and eventual apoptosis. The feces of elderly bats contain exceptionally high levels of an extremely potent IDO inhibitor, 3-amino-2-naphthoic acid [42]. Presence of this metabolite is rare and typically considered to be of synthetic, rather than biological, origin; although it has been recovered from potatoes and lemon extract [96,97]. While the exact origin of this compound in bat feces is unknown, it could be that 3-amino-2-naphthoic acid is a product of bat metabolism or the bat-specific microbiome. It could be that bats experience inhibition of the kynurenine pathway via inhibition of the enzyme IDO by 3-amino-2-naphthoic acid. If this pathway is in fact suppressed in elderly bats, our results would accordingly suggest that, with age, the activity of immune cells increases with age, making the elderly less susceptible to chronic inflammation and cancer.

### Efficacy of evidence obtained by fecal metabolomic assays

The exceptional longevity of bats suggests they might be an ideal model for the study of mammalian senescence. Unfortunately, studies to that effect are hindered in adult animals by the lack of visible age-related physiological changes and the lack of accurate methods of age determination. Here, we employed a non-invasive, fecal metabolomics approach to examine age-related differences in the fecal metabolomes of long-lived big brown bats (*Eptesicus fuscus*). Results establish the feasibility of this metabolomics approach and lay the foundation for future studies to expand our knowledge of age-related changes in bat fecal metabolic profiles and further develop fecal metabolomics as a means of age determination in bats. Our metabolomics approach, utilizing LC-QTOF-MS, identified at least 40 metabolites that were significantly increased in fecal samples of elderly *Eptesicus* over 14 years of age. While most of the candidate metabolites identified have known physiological significance in mammals (Figs 2 and 3), fourteen, as-yet unknown compounds, were also detected that demonstrated significant differences between our two age classes. Their exact origins, identity, and/or physiological relevance cannot be confirmed in this study and future work is necessary to identify these compounds.

A LC-QTOF-MS approach is ideal for rapidly and accurately quantifying large numbers of samples as it allows for almost real-time monitoring of concentrations of metabolites within 12–15 minutes. Once acquired, these metabolites can be identified using publicly available databases containing over 200,000 known compounds and, once identified, the physiological significance can be ascertained. Our non-targeted approach generated a list of metabolites that demonstrated significant variation between young and elderly animals. Although this study was limited by the necessity of examining age-related metabolomic changes in captive bats, future work is planned to replicate this study on a larger sampling of wild-caught bats that consume a uniform diet, as well as wild-caught bats that consume a natural diet. Future analyses examining metabolite differences between *ad libitum* captive diets and wild diets will allow us to address the effects of caloric restriction on age-related differences in fecal metabolic profiles.

If researchers lack access to an LC-QTOF-MS, results of this analysis may still be of interest. Within the metabolome of elderly bats, indole was found in high concentrations compared to younger animals. It could be that examining age-related changes in indole concentration may offer a means of estimating age groups in *Eptesicus*. A recent study described methods utilizing a spectrophotometer to quantify concentrations of indole within feces [44] and future work will quantify fecal indole concentrations in captive, known-aged and wild-caught unknown-aged *Eptesicus*. Because specimen preparation is rapid, bats may be briefly held in captivity to allow testing and then unneeded animals can then be released rather than subjected to invasive or fatal tests of age determination. Although quantification of indole alone is not as informative as broad-scale LC-QTOF-MS analyses, it may provide a valuable data point and allow for non-invasive age group estimation in *Eptesicus*. This study demonstrates non-invasive fecal metabolomics can be utilized to identify and compare differences in the fecal metabolic profiles of young and elderly bats and establishes fecal metabolomics as a promising method to discover unique adaptations to combat age-related declines in exceptionally long-lived mammals.

## Supporting information

S1 Table (XLSX)
